# Functionalization of Fe_3_O_4_ NPs by Silanization: Use of Amine (APTES) and Thiol (MPTMS) Silanes and Their Physical Characterization

**DOI:** 10.3390/ma9100826

**Published:** 2016-10-12

**Authors:** Silvia Villa, Paola Riani, Federico Locardi, Fabio Canepa

**Affiliations:** 1 Dipartimento di Chimica e Chimica Industriale, Università degli Studi di Genova, Via Dodecaneso 31, 16146 Genova, Italy; silvia.villa@chimica.unige.it (S.V.); federico.locardi@unige.it (F.L.); 2 Dipartimento di Chimica e Chimica Industriale, Università degli Studi di Genova and CNR-SPIN Genova Unit, Via Dodecaneso 31, 16146 Genova, Italy; fabio.canepa@unige.it

**Keywords:** magnetite nanoparticles, silica shell, AC magnetic susceptibility, functionalized organo silane molecules

## Abstract

In this paper the results concerning the synthesis of magnetite (Fe_3_O_4_) nanoparticles (NPs), their functionalization using silane derivatives, such as (3-Aminopropyl)triethoxysilane (APTES) and (3-mercaptopropyl)trimethoxysilane (MPTMS), and their exhaustive morphological and physical characterization by field emission scanning electron microscopy (FE-SEM) with energy dispersion X-ray spectrometer (EDX) analysis, AC magnetic susceptibility, UV-VIS and IR spectroscopy, and thermogravimetric (TGA) analyses are reported. Two different paths were adopted to achieve the desired functionalization: (1) the direct reaction between the functionalized organo-silane molecule and the surface of the magnetite nanoparticle; and (2) the use of an intermediate silica coating. Finally, the occurrence of both the functionalization with amino and thiol groups has been demonstrated by the reaction with ninhydrin and the capture of Au NPs, respectively.

## 1. Introduction

Magnetic nanoparticles (MNPs) have many attractive applications for a wide range of disciplines, including magnetic fluids, catalysis, biotechnology/biomedicine, magnetic resonance imaging, data storage, and environmental remediation [1,2,3,4,5,6,7,8,9,10,11,12]. While a number of suitable methods have been developed for the synthesis of magnetic nanoparticles, successful application is highly dependent on the stability of the particles under a range of different conditions.

However, among the different kinds of (MNPs), only maghemite (γ-Fe_2_O_3_) and magnetite (Fe_3_O_4_) NPs are mainly taken into account due to their lower toxicity for biomedical or environmental applications, with respect to other magnetic materials (Co- and Ni- based nanoparticles (NPs) [13]. Both iron oxides are ferrimagnetic with a magnetic moment of 2.5 μ_B_/f.u. (formulae units) for γ-Fe_2_O_3_ and 4.0 μ_B_/f.u. for Fe_3_O_4_, respectively [13]. Below a characteristic dimension (critical diameter *d_s_*), in single-domain magnetic nanoparticles, the magnetic behavior called superparamagnetism, is reached when the thermal energy is greater with respect to the anisotropy energy, so the magnetization vector can fluctuate easily between the up and down directions, with a time-averaged net moment equal to zero. The time required to have a change from the up to the down state is called *relaxation time* (τ_N_) and the temperature at which a change from the spin -flip free state to the blocked state is observed is called blocking temperature (*T*_B_). Since the time required to make a measurement is dependent on the equipment used, the blocking temperature for the same system of NPs could be different depending on the technique used. The superparamagnetic state is characterized by a zero value of the coercivity and this is the magnetic state commonly used for the above-cited applications.

Fe_3_O_4_ NPs are currently preferred to γ-Fe_2_O_3_ ones, due to larger magnetic moment and relatively simple synthetic methods. These NPs are also widely used as the starting point for complex functionalization in order to reach different properties. A typical “nano-architecture” is formed by a magnetic core plus one or more intermediate coatings before the final functionalization.

Usually, polymer or inorganic coatings are widely used when the core of functionalized “nano-architectures” needs to be protected from oxidation/corrosion processes, or when toxic or pollution problems, arising from the core, must be prevented. Furthermore, the use of inorganic structures (silica, alumina, or titania coatings) around the magnetic NPs increases their stability in solution, preventing or minimizing the formation of agglomerates due to magnetic dipole–magnetic dipole interactions. However, this step is always time consuming and is often quite expensive. Functionalized magnetic nanoparticles could be used to capture, for instance, toxic heavy metals or molecules, arising from relatively large quantities of waste water; this is a typical example where a fast and economic production of functionalized MNPs (without the presence of an intermediate coating) seems to be highly advisable.

The possibility of obtaining the same functionalization of the nanostructures with or without the presence of the intermediate coating is a desirable goal, but a comparison between the two complex nanosystems is fundamental to verify the achieved functionalization and its efficiency.

The purpose of the present paper is, thus, the functionalization of magnetite NPs with two different organo-silane molecules (OSM), namely (3-aminopropyl)triethoxysilane (APTES) and (3-mercaptopropyl)trimethoxysilane (MPTMS). Two different paths were used to achieve the desired functionalization: the first one is a direct reaction between the OSM and the surface of the magnetite nanoparticle (Fe_3_O_4_@OSM) and the second one is the use of an intermediate silica coating (using tetraethoxysilane (TEOS) as a reagent) with the silane derivative (Fe_3_O_4_@SiO_2_@OSM). The two paths are shown in the following:

Fe_3_O_4_ + OSM → Fe_3_O_4_@OSM

Fe_3_O_4_ + TEOS → Fe_3_O_4_@SiO_2_ + OSM → Fe_3_O_4_@SiO_2_@OSM

In order to verify the achieved functionalization and its efficiency, the different products have been analyzed by field emission scanning electron microscopy (FE-SEM) equipped with an energy dispersion X-ray spectrometer (EDX), IR spectroscopy, TGA (thermogravimetric analysis) and AC susceptibility measurements, adopting the physical model already proposed for a system of magnetic nanoparticles in suspension [14]. The presence of the primary amine of APTES on the surface of the functionalized NPs was confirmed by UV-VIS spectroscopy analyses using ninhydrin as the revealing agent [15]. The success of the thiol functionalization (MPTMS) was confirmed by the well-known reactivity of the -SH group with respect to Au. In fact Au NPs were successfully conjugated on the surface of Fe_3_O_4_@MPTMS and Fe_3_O_4_@SiO_2_@MPTMS, thus leading to a nanocomposite formed by a magnetic core of Fe_3_O_4_ surrounded by Au NPs.

We demonstrated here that Fe_3_O_4_@OSM particles could be used with an efficiency comparable to Fe_3_O_4_@SiO_2_@OSM ones, for all the applicative uses not requiring a high dispersion in a fluid, thus avoiding a further synthetic step.

## 2. Results and Discussion

### 2.1. APTES Functionalization

In this section the results concerning the two different paths used for the APTES functionalization are reported.

#### 2.1.1. FE-SEM Results

**Figure 1:**
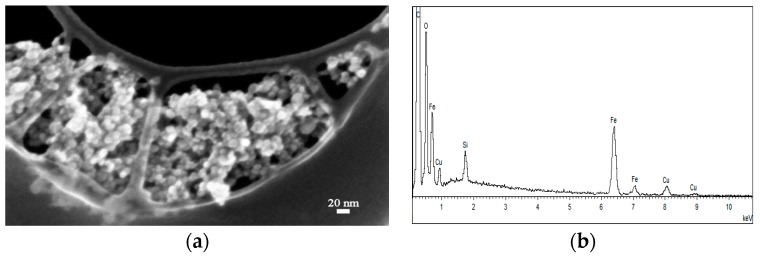
FE-SEM image of Fe_3_O_4_@APTES NPs (**a**); and the corresponding EDX spectrum (**b**). The Fe_3_O_4_@APTES NPs are laying on a carbon lace covering a copper grid support.

In Figure 1 the FE-SEM image of the Fe_3_O_4_@APTES NPs is shown with the corresponding EDX spectrum: the obtained microanalysis evidences the presence of Si related to the APTES functionalization. The Cu peaks come from the copper grids used as support. The image shows NPs organized in large agglomerates due to interparticle interactions. The mean radius measured on fifty nanoparticles belonging to an aggregate is about 7 nm.

**Figure 2:**
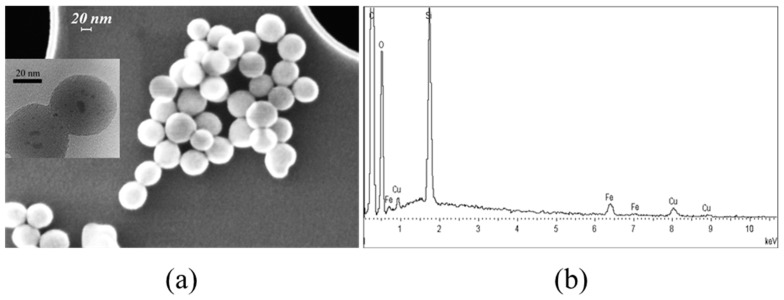
FE-SEM image of Fe_3_O_4_@SiO_2_@APTES NPs (**a**); and the corresponding EDX spectrum (**b**). In the inset of figure (**a**) a TEM image of the specimen is shown.

In Figure 2 the FE-SEM image of the Fe_3_O_4_@SiO_2_@APTES NPs is presented: it is evident that the better dispersion of these NPs, presenting a regular spherical shape, with dimensions between 40 and 100 nm. In the inset of Figure 2a, a TEM image of the same NPs is presented, showing the dark Fe_3_O_4_ NPs surrounded by large silica shells.

#### 2.1.2. UV-VIS Results

**Figure 3:**
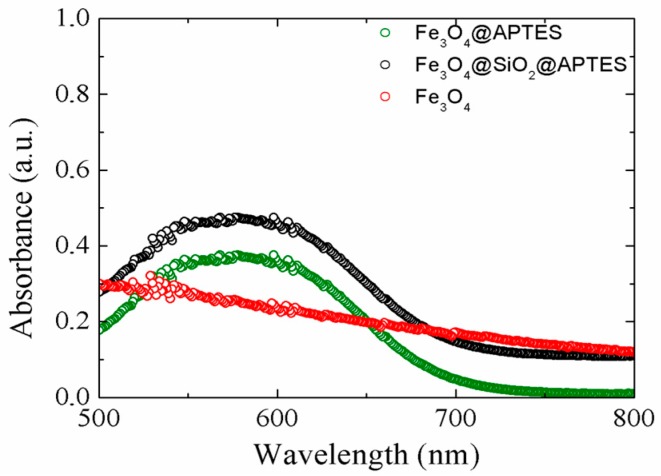
UV-VIS spectra of Fe_3_O_4_, Fe_3_O_4_@APTES, and Fe_3_O_4_@SiO_2_@APTES in ninhydrin/ethanol solution, red, green, and black curves, respectively. Green and black curves are superimposed, thus, in the graph, the corresponding spectra have been shifted.

In Figure 3, the UV-VIS spectra of Fe_3_O_4_@APTES and Fe_3_O_4_@SiO_2_@APTES treated with a ninhydrin/ethanol solution are presented. In both samples APTES molecules bonded on the surface of the NPs were detected by the appearance of the typical absorbance peak related to the formation of the Ruhemann complex around 560 nm [16], absent in the Fe_3_O_4_ alcoholic solution.

#### 2.1.3. Magnetic Results

Previous SQUID DC magnetic measurements performed on Fe_3_O_4_ NPs prepared in our laboratories [17] gave evidence that the adopted protocol results in nanoparticles with diameters between 3 and 7 nm, i.e., well below the superparamagnetic limit of 12.5 nm calculated for magnetite NPs (magnetocrystalline anisotropy constant Ku = 1.9 × 10^5^ J/m^3^ [18]). The room temperature isothermal magnetization of the bare Fe_3_O_4_ NPs displaying the absence of the coercivity, typical of the superparamagnetic regime, is shown in Figure S1 in the Supplementary Information.

Furthermore, we optimized an AC susceptibility model [14], based on the well-known Debye’s model for the electrical susceptibility of polar molecules [19], for magnetic nanoparticles in solution, where both Brownian relaxation (due to the coherent rotation of hydrated NPs in solution with the AC field) and Néel relaxation (due to the rotation of the magnetization vector of the NP with the alternating magnetic field) are taken into account. In our model, the real (*χ*′) and imaginary (*χ*″) components of the AC susceptibility as a function of the frequency *ν* are defined as:

$$\chi \prime \left(\nu \right)=\chi_{\infty }+\int_{r_{1}}^{r_{2}}\frac{\left(\chi_{0B}-\chi_{\infty }\right)\cdot P_{B}\left(r\right)}{1+{\left(2\pi \cdot \nu \cdot \frac{4\cdot \pi \cdot \eta }{k_{B}\cdot T}\cdot r_{hydr.}^{3}\right)}^{2}}dr+\int_{r_{1}}^{r_{2}}\frac{\left(\chi_{0N}-\chi_{\infty }\right)\cdot P_{N}\left(r\right)}{1+{\left(2\pi \cdot \nu \cdot {exp}^{\frac{4\cdot \pi \cdot K_{a}\cdot r_{c}^{3}}{3\cdot k_{B}\cdot T}}\right)}^{2}}dr$$

$$\chi^{\prime \prime }\left(\nu \right)=\int_{r_{1}}^{r_{2}}\frac{\left(\chi_{0B}-\chi_{\infty }\right)\cdot P_{B}\left(r\right)\cdot \left(2\pi \cdot \nu \cdot \frac{4\cdot \pi \cdot \eta }{k_{B}\cdot T}\cdot r_{hydr.}^{3}\right)}{1+{\left(2\pi \cdot \nu \cdot \frac{4\cdot \pi \cdot \eta }{k_{B}\cdot T}\cdot r_{hydr.}^{3}\right)}^{2}}dr+\int_{r_{1}}^{r_{2}}\frac{\left(\chi_{0N}-\chi_{\infty }\right)\cdot P_{N}\left(r\right)\cdot \left(2\pi \cdot \nu \cdot {exp}^{\frac{4\cdot \pi \cdot K_{a}\cdot r_{c}^{3}}{3\cdot k_{B}\cdot T}}\right)}{1+{\left(2\pi \cdot \nu \cdot {exp}^{\frac{4\cdot \pi \cdot K_{a}\cdot r_{c}^{3}}{3\cdot k_{B}\cdot T}}\right)}^{2}}dr$$

where *χ*_∞_ is the susceptibility at the highest frequency, *χ*_0*B*_ and *χ*_0*N*_ are the two contributions to the experimental susceptibility at zero frequency, *P_B_*(*r*) and *P_N_*(*r*) are the two log-normal Brownian and Néel size distribution functions, respectively, *η* is the viscosity of the solvent, *T* is the absolute temperature, *r_hydr._* is the hydrodynamic radius of the nanoparticle, *r_c_* the magnetic radius of the nanoparticle, and *K_a_* is the first order anisotropy constant. The correlation of the model to the experimental data is satisfied if, simultaneously, both components of the complex susceptibility are well fitted.

**Figure 4:**
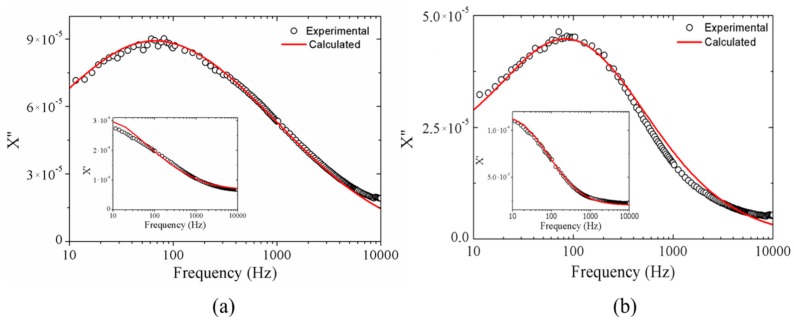
Imaginary and real (inset) components of the complex susceptibility of Fe_3_O_4_@APTES (**a**); and Fe_3_O_4_@SiO_2_@APTES (**b**). The symbols are the experimental points, while the red line is the fit with the model of [14].

In Figure 4 the complex susceptibility of a Fe_3_O_4_@APTES (a) and of Fe_3_O_4_@SiO_2_@APTES (b) samples are reported. In both plots the symbols represent the experimental data while the continuous red line represents the fit. In Table 1 the fit results for both systems are reported.

The values of the Brownian distribution mean radius for both systems are related to the stronger agglomeration among the magnetic nanoparticles for Fe_3_O_4_@APTES NPs with respect to the Fe_3_O_4_@SiO_2_@APTES ones. Moreover, comparing this result with the data from FE-SEM analysis (diameters between 40 and 100 nm) we can conclude that Fe_3_O_4_@SiO_2_@APTES NPs in the solvent are organized in small agglomerates formed by a few particles.

Finally, we want to emphasize here the complete agreement, as expected, between the results of the Néel distribution obtained for both coated and uncoated systems. We demonstrated, in our previous papers that the synthetic protocol used allows magnetite NPs with a mean diameter around 6–8 nm [17] to be obtained. This value is also confirmed by the magnetic analysis on the functionalized structures. 

#### 2.1.4. FT-IR Spectroscopy

**Figure 5:**
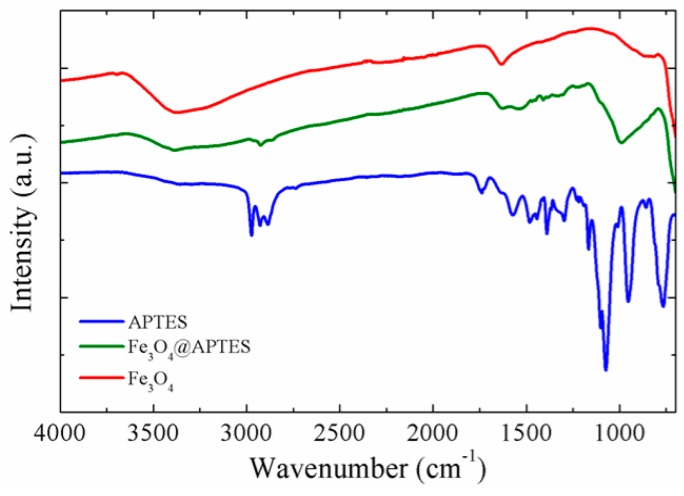
FT-IR spectra of bare Fe_3_O_4_ (red curve), Fe_3_O_4_@APTES (green curve), and pure APTES (blue curve).

To further investigate the nature of the final product, the dried powders have been analyzed by FT-IR measurement. Figure 5 reports the spectra collected on Fe_3_O_4_@APTES sample compared with bare Fe_3_O_4_ NPS and pure APTES.

In the figure the presence of peaks around 3000 cm^−1^, related to C–H stretching, can be clearly observed for the functionalized sample (green curve). The broad peak in the spectrum of bare Fe_3_O_4_ NPs at about 3400 cm^−1^ is due to O–H stretching. Furthermore, the functionalization causes the appearance of additional barely resolvable peaks in the fingerprint region. The strong peaks near 1100 and 800 cm^−1^ are due to Si–O–Si and Si–O stretching vibrations, respectively, which confirm the condensation between hydroxyl groups on the magnetite NPs’ surface and the alkoxysilane molecule. Thus, FT-IR measurements confirm the functionalization with organic moieties for the directly-functionalized particle.

Infrared analysis has also been performed for the silica coated samples; nevertheless, in this second case, the presence of silica did not allow to detect any other functional group.

#### 2.1.5. Thermogravimetric Analyses

In Figure S2 the TGA analysis of sample Fe_3_O_4_@SiO_2_@APTES is reported. Two different weight losses are identifiable. The first one, from low temperature up to 200 °C, is due to the release of the solvent absorbed onto the nanoparticle’s surface. This phenomenon is well known; in fact, as reported in the literature, small molecules, like water and ethanol, can be easily entrapped in porous matrices [20]. The second one started immediately after the aforementioned loss, and it is marked by a continuous decrease of sample weight until about 950 °C. In this range the organic ligand decomposes. The real degradation mechanism is not a trivial problem; moreover, literature data are very poor and not exhaustive [21]. The most reasonable hypothesis supposes the Si–C bond breaking, with the release of the organic chain, while the silane part of the ligand is still linked to the particle’s surface. The total weight loss amounts to 8.2%. For the magnetite sample Fe_3_O_4_@APTES (Figure S3), the decomposition of the organic ligand seems to follow a different mechanism characterized by a first decomposition step between 200 °C and 500 °C, and a second one in the 500–800 °C range. For this latter sample the total weight loss is about 7.8%.

### 2.2. MPTMS Functionalization

This section presents the results related to the thiol functionalization.

#### 2.2.1. FE-SEM Results

**Figure 6:**
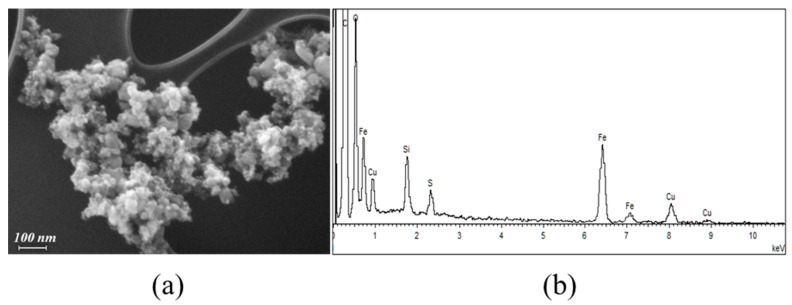
FE-SEM image of Fe_3_O_4_@MPTMS NPs (**a**); and the corresponding EDX spectrum (**b**).

In Figure 6a FE-SEM image of the magnetite NPs coated by the thiol derivative of the OSM, together with the EDX analysis, is shown. Additionally, in this case, the lack of a thick coating promotes magnetic dipole interactions, giving rise to large agglomerates. The presence of the thiol is confirmed by the EDX spectrum, where two small peaks related to the presence of sulphur can be observed. Furthermore, in this case, the mean radius measured on fifty NPs belonging to an aggregate is about 7 nm.

**Figure 7:**
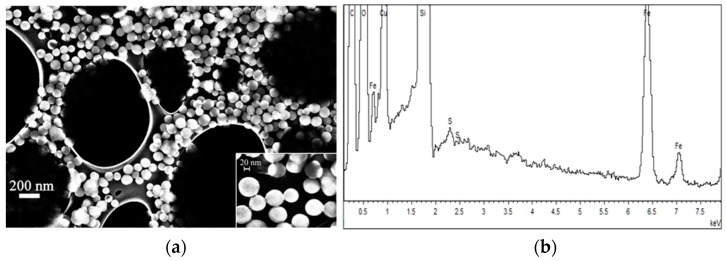
FE-SEM image of Fe_3_O_4_@SiO_2_@MPTMS NPs (**a**); and the corresponding EDX spectrum (**b**).

In Figure 7 an FE-SEM image related to the functionalized Fe_3_O_4_@SiO_2_@MPTMS NPs and its EDX analysis are presented. Here, the nanoparticles are well-defined and separated, with dimensions ranging from 50–110 nm. The EDX analysis displays, together with the peaks of Fe, Si, and O, two very small peaks related to sulphur, confirming again the functionalization with MPTMS.

#### 2.2.2. Magnetic Results

**Figure 8:**
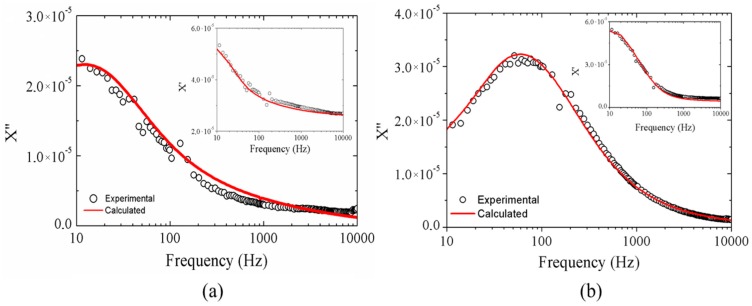
Imaginary and real, as inset, components of the complex susceptibility of Fe_3_O_4_@MPTMS (**a**); and Fe_3_O_4_@SiO_2_@MPTMS (**b**). The symbols are the experimental points, while the red line is the fit with the model of [14].

Figure 8 presents the AC susceptibility and the fit of Fe_3_O_4_@MPTMS (a) and Fe_3_O_4_@SiO_2_@MPTMS (b). The results of the fit are reported in Table 2.

Again, the values of the mean Brownian radius for both types of functionalized nanoparticles are related to very different morphologies: in Fe_3_O_4_@MPTMS we observe agglomerates due to strong magnetic dipole interactions, while the large silica shell is the major responsible of the Brownian mean radius of Fe_3_O_4_@SiO_2_@MPTMS. The magnetic results confirm, also in this case, that Fe_3_O_4_@SiO_2_@MPTMS particles are organized in small agglomerates formed by few particles in the solvent.

The contributions of the Néel distribution for both of the systems of MPTMS functionalized nanoparticles are comparable and in complete agreement with the information arising from the NPs functionalized with APTES, and with our already-presented results.

#### 2.2.3. FT-IR Spectroscopy

**Figure 9:**
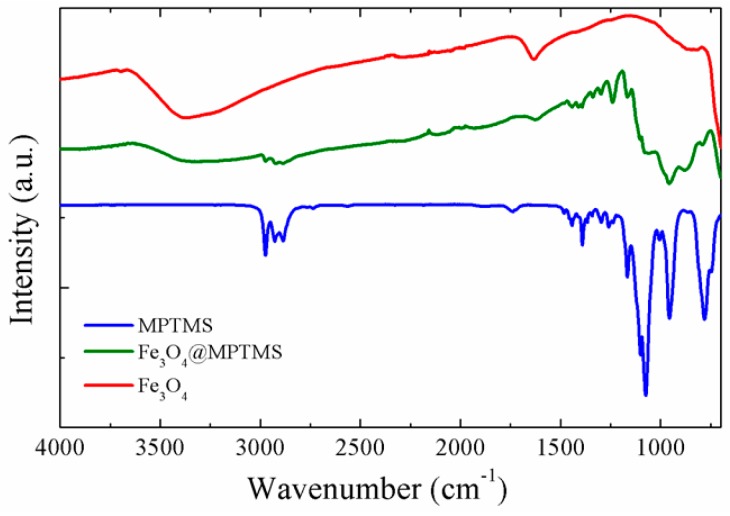
FT-IR spectra of bare Fe_3_O_4_ (red curve), Fe_3_O_4_@MPTMS (green curve), and pure MPTMS (blue curve).

To further investigate the nature of the final product, the dried powders have been analyzed by FT-IR measurements. Figure 9 reports the spectra collected on a Fe_3_O_4_@MPTMS sample compared with bare Fe_3_O_4_ NPS and pure MPTMS.

The spectra of both functionalized samples with APTES or MPTMS are very similar. In fact, in the Fe_3_O_4_@MPTMS spectrum (green curve), the peaks around 3000 cm^−1^, also related to C–H stretching, can be clearly observed. The broad peak in the spectrum of bare Fe_3_O_4_ NPs at about 3400 cm^−1^ is due to O–H stretching. Furthermore, the functionalization causes the appearance of additional barely-resolvable peaks in the fingerprint region. The strong peaks near 1100 and 800 cm^−1^ are due to Si–O–Si and Si–O stretching vibrations, respectively, which confirm the condensation between hydroxyl groups on magnetite NP surfaces and the alkoxysilane molecule. The typical, very weak, peak related to the S–H bond was observed only in the spectrum of the pure silane at about 2600 cm^−1^. Thus, FT-IR measurements confirm the functionalization with organic moieties for the directly-functionalized particle.

Infrared analysis has been also performed for the silica coated samples; nevertheless, in this second case, the presence of silica did not allow the detection of any other functional group.

#### 2.2.4. Thermogravimetric Analyses

**Figure 10:**
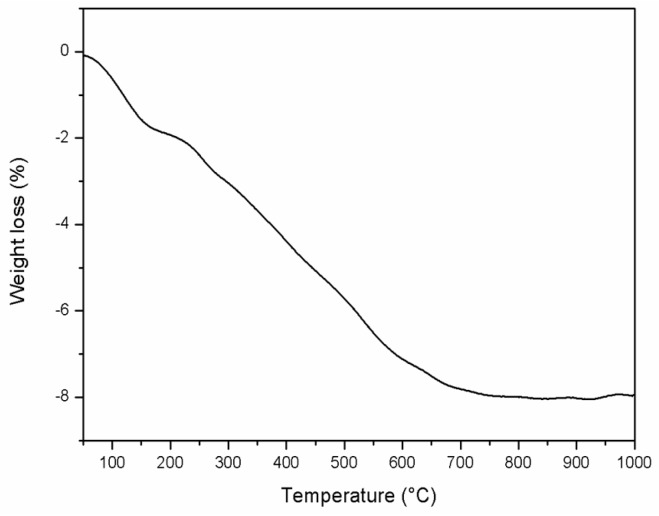
TGA analysis of Fe_3_O_4_@SiO_2_@MPTMS.

In Figure 10 and Figure S4 the TGA analyses of Fe_3_O_4_@SiO_2_@MPTMS and Fe_3_O_4_@MPTMS samples are reported. No significant differences were detected. The total weight loss amounted to 7.9% and 7.2%, respectively.

It should be noted that, for both APTES and MPTMS derivatives, the weight loss for the nanoparticles with the silica intermediate coating is a slightly higher with respect to those without any silica intermediate, in agreement with the trend reported by Čampelj et al. [22]. In any case these results also confirmed that the amount of organic ligands bonded to the inorganic surface of all of the analyzed nanostructures, could be considered comparable. Moreover, in this way we demonstrate that the synthetic processes give good results in both cases; thus, the use of the inorganic coating could be strictly required only for specific uses, for instance for biomedical applications.

#### 2.2.5. Au NP Conjugation

**Figure 11:**
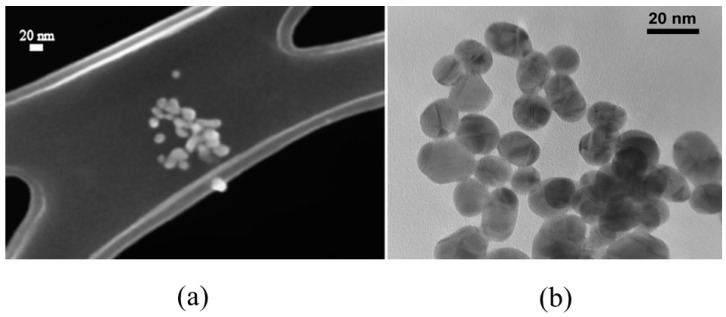
FE-SEM (**a**) and TEM (**b**) images of Au NPs.

Gold NPs have been synthetized following the synthetic method reported by Turkevich et al. [23]. A characteristic image of the synthesized Au NPs is shown in Figure 11. From different images of these NPs a mean diameter ranging from 8 to 20 nm has been evaluated.

These data are also confirmed by UV-VIS measurements (see Figure S5b): the observed absorbance peak at 524 nm is related to a gold particle dimension of about 20 nm, as reported in the literature [23], in excellent agreement with the FE-SEM observation.

**Figure 12:**
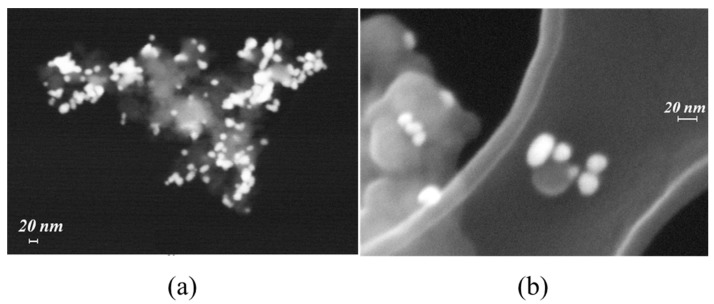
FE-SEM images of Fe_3_O_4_@MPTMS@Au (**a**) and Fe_3_O_4_@SiO_2_@MPTMS@Au (**b**) NPs.

Figure 12 has been collected using the back-scattered signal since it allows for obtaining a better contrast for different Z-value materials. In these conditions, Au NPs appear brighter than magnetite NPs. The Fe_3_O_4_@MPTMS@Au sample (Figure 12a) is characterized by strong agglomeration. In this sample, Au NPs are randomly distributed across the surface of the agglomerates. Furthermore, all of the detected Au NPs have been found linked to a magnetic moiety; no free Au NPs were observed. Figure 12b refers to a Fe_3_O_4_@SiO_2_@MPTMS@Au sample. Additionally, in this case, Au NPs can be found linked to the Fe_3_O_4_@SiO_2_@MPTMS surface. Since the agglomeration of the sample is much lower with respect to the non-stabilized one, single-coated particles with Au on the surface can be detected. Again, no free Au NPs have been observed.

**Figure 13:**
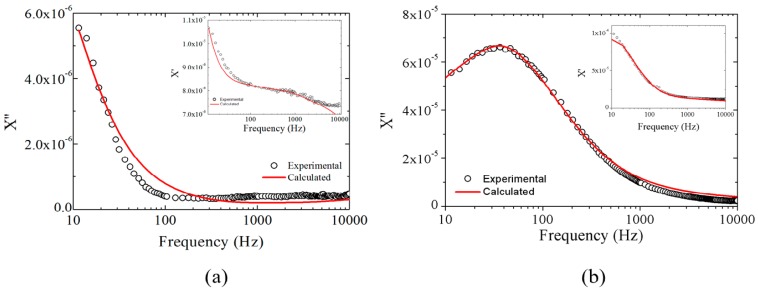
Imaginary and real, as inset, components of the complex susceptibility of Fe_3_O_4_@MPTMS@Au (**a**); and Fe_3_O_4_@SiO_2_@MPTMS@Au (**b**). The symbols are the experimental points, while the red line is the fit with the model of ref [14].

Figure 13 displays the AC susceptibilities of Fe_3_O_4_@MPTMS@Au (a) and Fe_3_O_4_@SiO_2_@MPTMS@Au (b) NPs, while the results of the fits are presented in Table 3. From Figure 13a a maximum centered below 10 Hz can be observed. The fit results give evidence of Fe_3_O_4_@MPTMS@Au agglomerates with relatively large dimensions of r_m_ = 210 nm. For Fe_3_O_4_@SiO_2_@MPTMS@Au (Figure 13b) a well-defined maximum is observed. The increase of the mean radius from 95 nm for Fe_3_O_4_@SiO_2_@MPTMS NPs to 115 nm for Fe_3_O_4_@SiO_2_@MPTMS@Au NPs is totally ascribed to the mean diameter of the gold nanoparticles, as shown in the image reported in Figure 12b.

## 3. Materials and Methods

### 3.1. Materials and Synthetic Method

All chemicals (99.9 wt % purity minimum) were purchased from Sigma Aldrich (St. Louis, MO, USA) and used without further purification.

Magnetite nanoparticles were synthesized using a modified Massart method [24] based on the co-precipitation of stoichiometric iron (II) and iron (III) chloride hexa-hydrate salts in aqueous ammonia solution. The obtained nanoparticles were washed several times in hot water and magnetically collected, following the protocol previously described [17]. The NPs were finally dispersed in water to be used for further functionalization.

Silica-coated magnetite NPs were prepared following the Stöber process [25]. Briefly, to an alcoholic suspension of magnetite (60 mg/L), water, ammonia, and TEOS are added. The reaction was then maintained for two hours at 40 °C and, finally, the product is magnetically collected. Details about the synthesis were already presented [26].

**Scheme 1:**
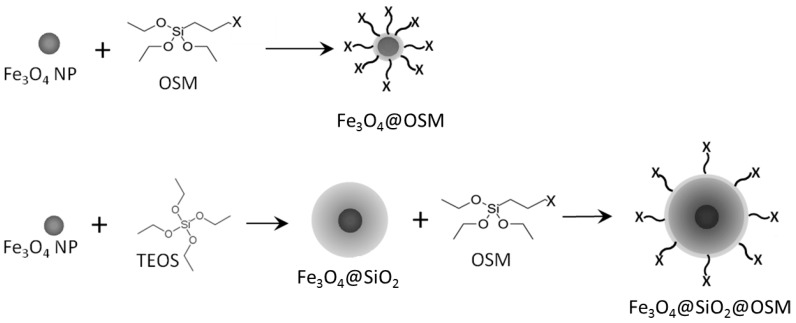
The different paths used to synthesize magnetite NPs functionalized with the organo-silane molecules.

The two different paths used to functionalize the magnetite NPs are reported in Scheme 1, where OSM is the generic organo-silane molecule and X is the functional group (–NH_2_ or –SH).

#### 3.1.1. APTES Functionalization

The protocol used for the direct APTES [(3-Aminopropyl)triethoxysilane] functionalization of magnetite nanoparticles is described here: 40 mL of a dispersion of Fe_3_O_4_ NPs in water (2 g/L) were added to 40 mL of ethanol and to 1.6 mL of 2% v/v solution of APTES. The temperature was kept constant at 50 °C and the reaction was carried out for 24 h.

The very low concentration of silica coated magnetite nanoparticles suggested the use of smaller volumes for reactants: 1 mL of Fe_3_O_4_@SiO_2_ (60 mg/L) in water was added to 1 mL ethanol and to 43 μL of APTES (2% v/v); temperature and time were the same as in the previous functionalization [27].

Each sample has been magnetically washed several times using first ethanol and then Milli-Q water (MilliQ Academic from Merck Millipore, Darmstadt, Germany).

#### 3.1.2. MPTMS Functionalization

The method for the direct functionalization of Fe_3_O_4_ NPs with MPTMS [(3-mercaptopropyl) trimethoxysilane] is reported here: 40 mL of a dispersion of magnetite NPs in water (0.5 g/L) were added to 40 mL of ethanol and 1.6 mL of MPTMS (2% v/v). The reaction was carried out in the same conditions reported above.

The standard reaction used to functionalize silica-coated magnetite NPs is: to 1 mL of ethanol in Eppendorf, 1 mL of Fe_3_O_4_@SiO_2_ NPs (60 mg/L) in water, and 20 μL of a solution 1% v/v of MPTMS and 11.5 μL of acetic acid were added. The reaction was carried out in the same conditions reported above [28]. As for APTES-functionalized samples, each sample has been magnetically washed several times using ethanol and then Milli-Q water.

#### 3.1.3. Synthesis of Au NPs

Gold NPs were prepared by the reduction of an aqueous solution of HAuCl_4_ (0.01 M) with sodium citrate (0.01 M) in a ratio of 1:3 (v/v) at room temperature under vigorous sonication for 15 min. After a few minutes, a color change from yellow to purple is easily detectable: the solution so obtained is stable for a very long time and does not require further treatments. A characteristic image of the synthesized Au NPs is shown in Figure 10.

#### 3.1.4. MPTMS-Au Conjugation

The gold NP conjugation to NPs@MPTMS (Fe_3_O_4_@MPTMS and Fe_3_O_4_@SiO_2_@MPTMS) (1:1 wt/wt ratio) is performed at RT in Eppendorf under vigorous mechanical stirring for 24 h. The NPs are then washed three times in Milli-Q water to completely eliminate the chemical environment, and then magnetically separated.

### 3.2. Characterization Techniques

Field emission scanning electron microscopy (FE-SEM) analyses were carried out using a FE-SEM JEOL ZEISS SUPRA 40 VP (Carl Zeiss AG, Oberkochen, Germany). Transmission electron microscopy (TEM) analyses were performed with the model JEOL JEM 2010 200 kV microscope from JEOL Ltd. (Peabody, MA, USA). The specimen powders for FE-SEM and TEM analyses were suspended in ethanol, and then exposed to ultrasonic vibrations to decrease the aggregation. A drop of the resultant mixture was deposited on a copper grid covered with a thin carbon lace and then carefully dried before the analysis.

Information on the organic molecules linked to the surface of the particles have been obtained by means of FT-IR spectrometry. For our purpose, we investigated dried samples with a Perkin Elmer Spectrum 65 FT-IR spectrometer (Perkin Elmer, Waltham, MA, USA) equipped with universal ATR (Attenuated Total Reflectance) sampling accessory.

Gold particle dimensions have been evaluated by means of FE-SEM and TEM observation, and indirectly determined by UV measurements of the absorbance in 0.01 M aqueous solution, using a Varian CARY50 UV-VIS spectrometer (Varian Medical Systems, Palo Alto, CA, USA). The organic compound ninhydrin (2,2-dihydroxyindane-1,3-dione) was used as the revealing agent. This chemical, when reacting with primary or secondary amines, produces a deep blue or purple color known as a Ruhemann complex [16].

AC susceptibility measurements were obtained using an Oxford Maglab2000 (Oxford Instruments, Abingdon on Thames, UK), operating in the 1–10^4^ Hz frequency range with 10 Oe applied a.c. magnetic field. The resolution of the AC signal was better than 10^−7^ emu.

Thermogravimetric analyses (TGA) were performed using a Labsys EVO Setaram instrument (Setaram Instrumentation, Caluire, France). Approximately 5 mg of each sample were weighed in an open alumina crucible (Setaram Instrumentation, Caluire, France) and heated from 50 °C to 1000 °C in a He atmosphere (20 mL/min) with a heating rate equal to 10 °C/min.

## 4. Conclusions

In this paper the results of the functionalization of magnetite nanoparticles with organo-silane molecules (APTES and MPTMS) using two different paths have been presented.

The functionalized magnetic NPs have been analyzed by different experimental techniques and the results are completely in agreement with each other. Furthermore, the magnetic susceptibility confirms the ability to achieve dimensional information about systems of magnetic nanoparticles in solution.

We demonstrated here the possibility of obtaining the same functionalization on the nanoparticles’ surfaces with and without the presence of the intermediate coating. Thus, the Fe_3_O_4_@OSM particles could be used with an efficiency comparable to the Fe_3_O_4_@SiO_2_@OSM one, for all of the applicative uses, for instance in the analytical and/or environmental fields, not requiring a high dispersion in a fluid, thus avoiding a further synthetic step.

## Figures and Tables

**Table 1:** Mean Brownian and Néel radii, obtained from the analysis of the AC susceptibility data for the Fe_3_O_4_@APTES and Fe_3_O_4_@SiO_2_@APTES NPs; σ is the standard deviation of the distribution.

Sample	Brownian Distribution		Néel Distribution	
	r_m_ (nm)	σ	r_m_ (nm)	σ
Fe_3_O_4_@APTES	115	0.6	3.8	0.03
Fe_3_O_4_@SiO_2_@APTES	80	0.4	4	0.04

**Table 2:** Mean Brownian and Néel radii, obtained from the analysis of the AC susceptibility data for the Fe_3_O_4_@MPTMS and Fe_3_O_4_@SiO_2_@MPTMS NPs; σ is the standard deviation of the distribution.

Sample	Brownian Distribution		Néel Distribution	
	r_m_ (nm)	σ	r_m_ (nm)	σ
Fe_3_O_4_	18	0.55	3.4	0.4
Fe_3_O_4_@MPTMS	160	0.25	4	0.05
Fe_3_O_4_@SiO_2_	82	0.25	4	0.05
Fe_3_O_4_@SiO_2_@MPTMS	95	0.25	4	0.05

**Table 3:** Mean Brownian and Néel radii, obtained from the analysis of the AC susceptibility data for the Fe_3_O_4_@MPTMS@Au and Fe_3_O_4_@SiO_2_@MPTMS@Au NPs: σ is the standard deviation of the distribution.

Sample	Brownian Distribution		Néel Distribution	
	r_m_ (nm)	σ	r_m_ (nm)	σ
Fe_3_O_4_@MPTMS@Au	210	0.15	4	0.05
Fe_3_O_4_@SiO_2_@MPTMS@Au	115	0.3	4	0.1

## Supplementary Materials

The following are available online at www.mdpi.com/1996-1944/9/10/826/s1. Figure S1: Room temperature isothermal magnetization of the bare Fe_3_O_4_ NPs, displaying the absence of the coercivity, typical of the superparamagnetic regime, Figure S2: TGA analysis of Fe_3_O_4_@SiO_2_@APTES, Figure S3: TGA analysis of Fe_3_O_4_@APTES, Figure S4: TGA analysis of Fe_3_O_4_@MPTMS, Figure S5: FE-SEM image of Gold NPs (a) and the relative UV-VIS spectrum (b).
