# Supplementary material for: Functionalization of Fe3O4 NPs by Silanization: Use of Amine (APTES) and Thiol (MPTMS) Silanes and Their Physical Characterization
Source: Materials (Basel). 2016 Oct 12;9(10):826. doi: 10.3390/ma9100826 (PMC5456583; doi:10.3390/ma9100826)
Supplement: Supplementary file 1 [file materials-09-00826-s001.pdf]

Supplementary Materials: Functionalization of Fe_3O_4 NPs by Silanization: Use of Amine (APTES) and Thiol (MPTMS) Silanes and Their Physical Characterization

Silvia Villa, Paola Riani, Federico Locardi and Fabio Canepa

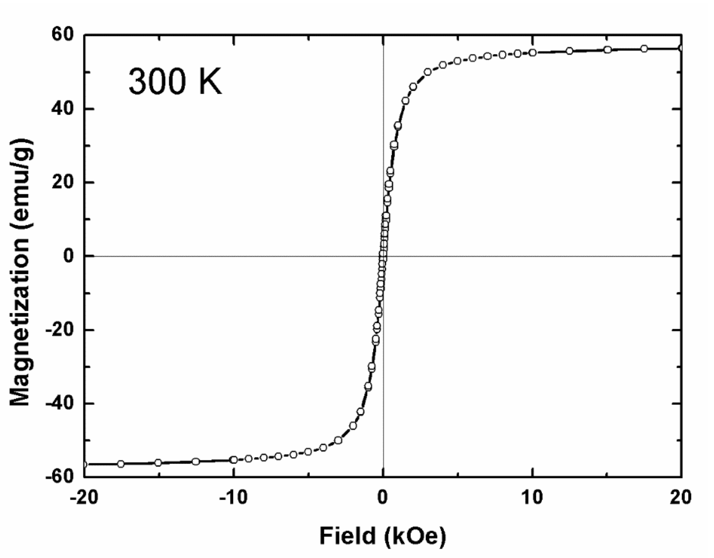

Figure S1. Room temperature isothermal magnetization of the bare Fe_3O_4 NPs, displaying the absence of the coercivity, typical of the superparamagnetic regime.

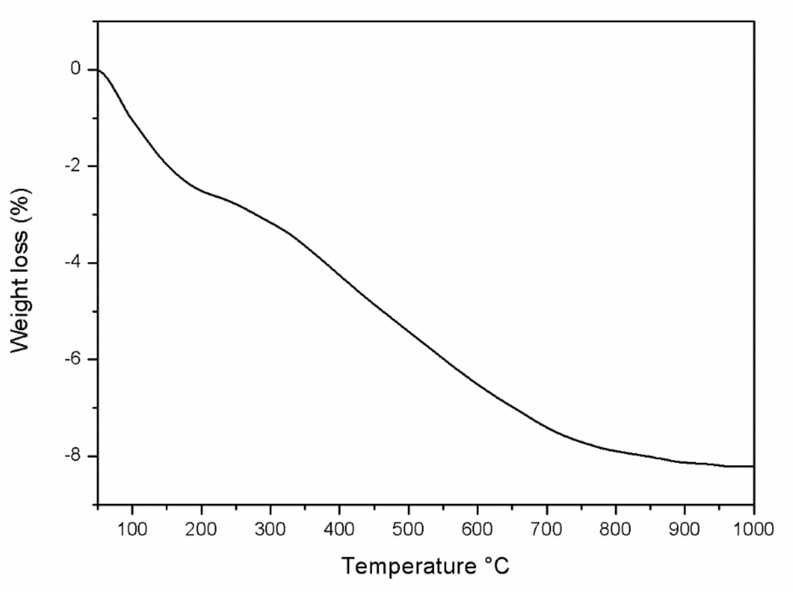

Figure S2. TGA analysis of $Fe_3O_4@SiO_2@APTES$.

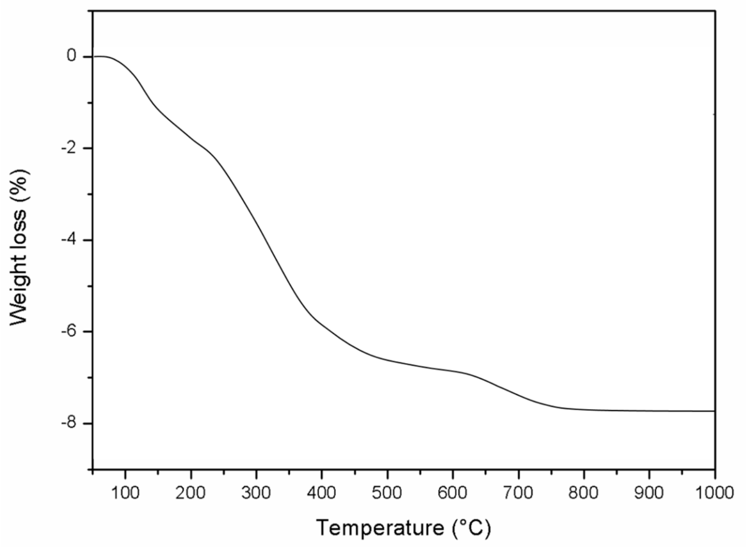

Figure S3. TGA analysis of Fe_3O_4@APTES.

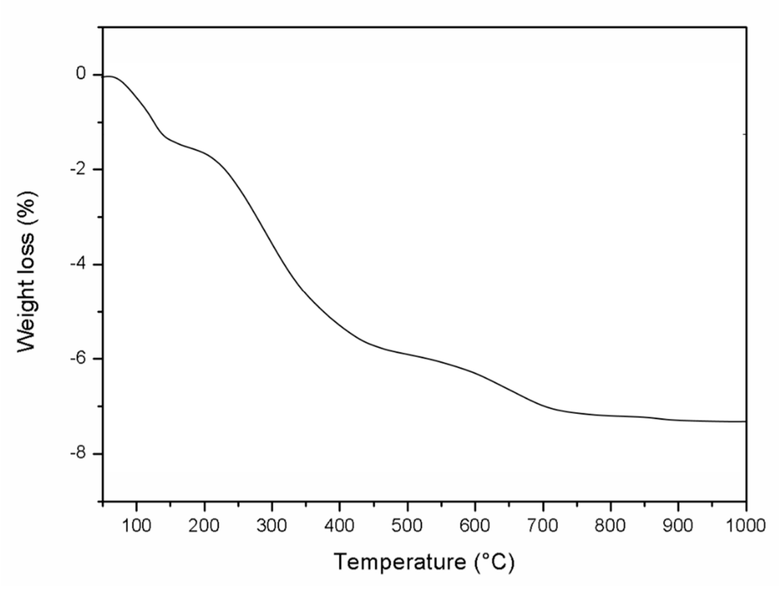

Figure S4. TGA analysis of Fe_3O_4@MPTMS.

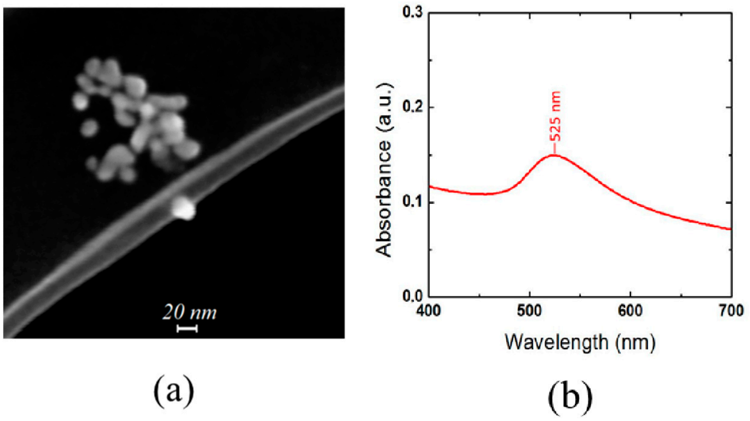

Figure S5. FE-SEM image of Gold NPs (**a**) and the relative UV-VIS spectrum (**b**).
